# Nocturnal hypoxemia in COPD: the amplifying effect of comorbid OSA and PLMS on oxygen desaturation

**DOI:** 10.1177/17534666251380431

**Published:** 2025-09-17

**Authors:** Viraj Jain, Harshill Modi, Moon Park, Anil Ghimire, Lourdes M. DelRosso

**Affiliations:** Loyola Marymount University, Los Angeles, CA, USA; University of California, San Francisco, Fresno, CA, USA; University of California, San Francisco, Fresno, CA, USA; University of California, San Francisco, Fresno, CA, USA; University of California, San Francisco, Fresno, CA, USA

**Keywords:** chronic obstructive, hypoxia, obstructive, periodic limb movement disorder, pulmonary disease, sleep apnea

## Abstract

**Background:**

Chronic obstructive pulmonary disease (COPD), obstructive sleep apnea (OSA), and periodic limb movements of sleep (PLMS) frequently co-occur and may exacerbate nocturnal hypoxemia. Still, their combined effects are not well defined.

**Objectives:**

To examine the independent and interactive effects of COPD, OSA, and PLMS on nocturnal oxygen desaturation.

**Design:**

Cross-sectional analysis of a sleep study cohort.

**Methods:**

We analyzed 711 participants (mean age 57.2 ± 17.9 years; 44.4% male), including 48 with COPD. Time with oxygen saturation ⩽88% (ST) and mean SpO_2_ were compared across COPD and OSA/PLMS subgroups. Multivariable regression tested the independent and interaction effects of COPD, OSA, PLMS, age, and sex.

**Results:**

Participants with COPD had lower mean SpO_2_ and longer ST than non-COPD participants (*p* < 0.005). ST was greatest in those with both OSA and PLMS, particularly in COPD. COPD (+46.4 min, *p* < 0.001) and OSA (+10.5 min, *p* = 0.009) independently increased ST. A negative COPD × OSA interaction (*p* = 0.021) indicated less-than-additive effects, whereas a positive COPD × OSA × PLMS interaction (*p* = 0.036) identified the highest desaturation burden.

**Conclusion:**

COPD and OSA independently worsen nocturnal hypoxemia, while the coexistence of COPD, OSA, and PLMS confers the greatest desaturation burden, underscoring the importance of evaluating overlapping conditions in clinical assessment.

## Plain language summary

Low nighttime oxygen in COPD: How sleep apnea and leg movements make it worse

People with chronic obstructive pulmonary disease (COPD) often have trouble getting enough oxygen during sleep, which can affect their health over time. This study looked at how two other common sleep problems—obstructive sleep apnea (OSA) and periodic limb movement disorder (PLMD)—might make this issue worse. We analyzed the sleep studies of over 700 adults, some with COPD and some without. We found that people with both COPD and either sleep apnea or frequent leg movements during sleep had more severe drops in their oxygen levels at night. Those with all three conditions, COPD, OSA, and PLMD, had the worst oxygen levels. This suggests that having multiple sleep disorders can combine to make nighttime breathing problems more serious in people with COPD. These findings highlight the importance of screening for and treating sleep disorders in people with COPD to protect their sleep quality and overall health.

## Introduction

Chronic obstructive pulmonary disease (COPD) is a common respiratory condition marked by long-term airway obstruction and inflammatory changes that worsen over time. COPD significantly impacts patients’ quality of life and is associated with a high burden of comorbidities, including cardiovascular disease, metabolic disorders, and sleep-related breathing disorders.^1^ Among these, sleep-disordered breathing, such as obstructive sleep apnea (OSA) and periodic limb movements of sleep (PLMS), has gained increasing attention due to its complex interactions with nocturnal hypoxemia and respiratory physiology in patients with COPD.^2^

Although sleep is essential for overall health, patients with COPD frequently experience disrupted sleep and low oxyhemoglobin saturation levels, which can intensify coexisting health problems.^3^ Patients with COPD frequently experience nocturnal hypoxemia, even in the absence of OSA, due to diminished ventilatory responses and impaired gas exchange. OSA can exacerbate this nocturnal hypoxemia with further disrupted sleep architecture and oxygenation.^4^ Saturation time (ST) below 88% is a clinically significant metric of nocturnal hypoxemia and is strongly associated with adverse outcomes, including increased cardiovascular risk and disease progression.

Reports indicate that between one-fifth and two-thirds of individuals with COPD also have OSA, a coexistence commonly termed the “overlap syndrome”^5^ Previous studies have explored the individual effects of OSA in patients with COPD, showing that patients with overlap syndrome tend to experience more severe symptoms than those with either condition alone.^5^ Many patients with OSA have elevated PLMS. PLMS occur more often in those with OSA, nearly 20%–50% depending on the study.^6,7^ While estimates for the general population are closer to 4%–11%.^8^ Few studies have directly assessed how PLMS, when combined with COPD and OSA, affect nocturnal oxygen levels and sleep architecture.

This study evaluates the differences in sleep architecture, oxygenation metrics, and respiratory indices between COPD and non-COPD patients. By stratifying participants into OSA/PLMS subgroups, we aim to delineate these conditions’ additive or synergistic effects. We hypothesize that COPD patients with both OSA and PLMS exhibit the worst nocturnal oxygenation compared to those with either condition or none.

## Methods

### Study design and participants

This observational, retrospective review examined consecutive full polysomnography (PSG) studies performed between June 2022 and July 2024 at the University of California, San Francisco (UCSF), Fresno Inspire Health Sleep Center, a tertiary referral sleep clinic. Inclusion criteria included consecutive full-night polysomnography on adult patients performed between June 2022 and July 2024. Exclusion criteria included PSGs performed for titration, split-night protocols, or those that were incomplete. The sleep center is under the division of Pulmonary (AG).

Medical records were reviewed to identify individuals with COPD. COPD was defined based on a physician‑documented diagnosis (post‑bronchodilator forced expiratory volume in 1 s to forced vital capacity ratio <0.70 together with compatible respiratory symptoms). Among 52 patients with COPD who underwent full PSG during the study period, four were excluded because their studies were split‑night or incomplete, leaving 48 COPD participants for analysis. These participants constituted the COPD 1 group. The comparison group consisted of 663 individuals without COPD (COPD 0).

**Figure 1.**
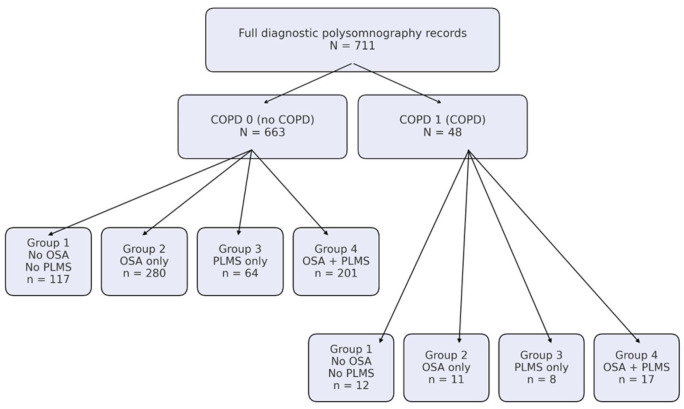
Participant flow diagram and subgroup allocation. CONSORT flow diagram illustrating participant selection and subgroup allocation. This CONSORT‑style flow diagram summarizes participant selection and allocation into COPD and OSA/PLMS subgroups. Beginning with 711 eligible polysomnography records, participants were categorized into non‑COPD (COPD 0, *n* = 663) and COPD (COPD 1, *n* = 48) groups. Each COPD status was further stratified into four subgroups based on the presence or absence of OSA and elevated PLMS (Group 1: no OSA/no PLMS; Group 2: OSA only; Group 3: PLMS only; Group 4: OSA + PLMS), with sample sizes indicated within the diagram. This flowchart clarifies the number of participants represented in each subgroup and highlights the small sample sizes within some COPD subgroups. COPD, chronic obstructive pulmonary disease; OSA, obstructive sleep apnea; PLMS, periodic limb movements of sleep.

Because all PSGs were performed for clinical indications, the apnea–hypopnea index (AHI) and periodic limb movement index (PLMI) were routinely measured for every participant. Patients with AHI ⩾ 5 events per hour were considered to have OSA. Participants with PLMI ⩾ 15 events per hour were classified as having elevated PLMS. PLMS were quantified, but a formal clinical diagnosis of periodic limb movement disorder (PLMD) requires PLMS associated with arousals and daytime symptoms; therefore, throughout this manuscript, we refer to the high‑PLMI group as the “PLMS” subgroup rather than PLMD. Participants were stratified into four subgroups: (1) no OSA/no PLMS, (2) OSA only, (3) PLMS only, and (4) OSA + PLMS. The study was approved by the Community Health System IRB 2023052. CONSORT Diagram is found in Figure 1.

### Polysomnography

Polysomnography was performed according to the American Academy of Sleep Medicine (AASM) criteria.^9^ The data were recorded using the Respironics Alice 6 system. Parameters recorded included electroencephalogram (EEG; two frontal, two central, and two occipital channels, referred to the contralateral mastoid); electrooculogram, electromyogram (EMG) of the submentalis muscle, EMG of the right and left tibialis anterior muscles, respiratory signals, effort signals for thorax and abdomen, oximetry, capnography, a single-lead electrocardiogram, video, and audio recording. Sleep technicians performed calibrations according to routine standards. Sleep stages, respiratory events, and PLMS were scored by a certified sleep technologist and board-certified sleep physician, according to the AASM criteria.^10^

### Statistical analysis

The dataset was analyzed using IBM SPSS 29.0.2. Summary statistics were calculated for all continuous variables and presented as mean ± standard deviation (SD) for normally distributed data or median and interquartile range (IQR) for skewed data. Categorical variables were expressed as absolute frequencies and percentages.

For primary comparisons, a two-sample *t*-test (or Mann–Whitney U test for non-normal data) was conducted to compare parameters between the COPD 1 and COPD 0 groups. Age-adjusted comparisons were performed using analysis of covariance (ANCOVA).

To evaluate the effects of OSA and PLMS on saturation time (ST ⩽ 88%), a two-way ANOVA was conducted to test main effects and interaction effects, stratified by COPD status. For subgroup analyses (groups 1–4: no OSA/PLMS, OSA only, PLMS only, OSA + PLMS), a one-way ANCOVA adjusting for age was performed, followed by post hoc Tukey’s tests for pairwise comparisons. In addition, multivariable linear regression was used to assess the independent effects of COPD, OSA, PLMS, age, and sex on ST ⩽88%.

The cumulative distribution of ST was visualized separately for the COPD 0 and COPD 1 groups. Within each COPD status, CDFs were stratified by the four OSA/PLMS groups to examine distributional differences.

All *p*-values were adjusted for multiple comparisons using Tukey’s method. A *p*-value < 0.05 was considered statistically significant.

A post hoc power analysis was conducted using the primary comparison of ST ⩽ 88% between the COPD 0 and COPD 1 groups. With 663 participants in COPD 0 and 48 in COPD 1, the study had > 99% power (α = 0.05) to detect the observed effect size (Cohen’s *d* = 0.94). The minimum detectable effect size with 80% power was *d* = 0.42 (moderate effect).

### Reporting

The reporting of this study conforms to the Strengthening the Reporting of Observational Studies in Epidemiology (STROBE) statement.^11^ The checklist from the relevant guideline can be found in the Supplemental Files.

## Results

The study included a total of 711 participants, with 48 participants with a diagnosis of COPD (COPD 1 group) and 663 participants without a diagnosis of COPD (COPD 0 group). The mean age of the participants was 57.2 .2 ± 17.9 9 years. Among the participants, 316 (44.4%) were males, and 395 (55.6%) were females. The ethnic distribution was as follows: White (380, 53.4%), Hispanic (224, 31.5%), Asian (47, 6.6%), Black (43, 6.0%), and Other (13, 1.8%; Table 1).

**Table 1.** Participant demographics.

Variable	Total (*N* = 711)	COPD 0 (*N* = 663)	COPD 1 (*N* = 48)
Age, mean ± SD (years)	57.2 ± 17.9	56.4 ± 18.0	68.6 ± 12.1
Sex, male	316 (44.4%)	298 (44.9%)	18 (37.5%)
White	380 (53.4%)	350 (52.8%)	30 (62.5%)
Hispanic	224 (31.5%)	215 (32.4%)	9 (18.8%)
Asian	47 (6.6%)	47 (7.1%)	0 (0.0%)
Black	43 (6.0%)	34 (5.1%)	9 (18.8%)
Other	13 (1.8%)	13 (2.0%)	0 (0.0%)

**Figure 2.**
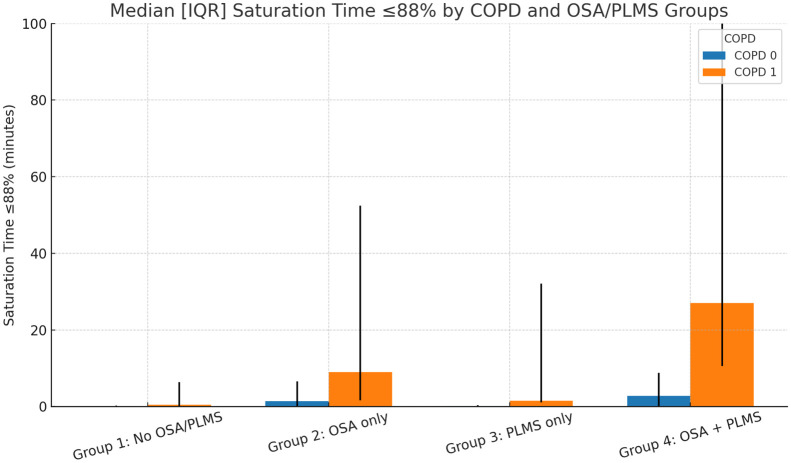
Saturation time spent below 88% by the OSA/PLMS subgroup and COPD status. Median (bars) and interquartile range (error bars) of saturation time ⩽88% are shown across OSA/PLMS subgroups, stratified by COPD status. Groups: Group 1 = No OSA/PLMS (COPD 0: *n* = 117; COPD 1: *n* = 12), Group 2 = OSA only (COPD 0: *n* = 280; COPD 1: *n* = 11), Group 3 = PLMS only (COPD 0: *n* = 64; COPD 1; *n* = 8), Group 4 = OSA + PLMS (COPD 0: *n* = 201; COPD 1: *n* = 17). COPD 0 = participants without COPD; COPD 1 = participants with COPD. The Y-axis is truncated at 100 min for visualization. COPD, chronic obstructive pulmonary disease; OSA, obstructive sleep apnea; PLMS, periodic limb movements of sleep.

When comparing patients with and without COPD, adjusting for age, most measures of sleep architecture and respiratory indices were similar, with the only statistically significant difference being the nadir oxygen saturation and time spent with saturation at or below 88% (ST) (Table 2). Differences emerged in measures of nocturnal oxygenation. Participants with COPD had lower mean SpO_2_ (91.1 ± 2.8% vs 93.3 ± 2.2%, *p* < 0.005) and longer time spent with saturation at or below 88% (ST ⩽ 88%). Median ST was 27.0 min (IQR 7.5–68.0) in COPD compared with 2.0 min (IQR 0.0–8.0) in non-COPD participants (*p* < 0.005). Among those with COPD, 12 had no OSA or PLMS (group 1), 11 had OSA alone (Group 2), 8 had PLMS alone (Group 3), and 17 had both OSA and PLMS (Group 4). ANCOVA adjusting for age showed significant differences for ST and other parameters. Post hoc tests indicated that ST was highest in group 4 (38.0 (18.0–79.0); *p* = 0.001, adjusted for multiple comparisons (Table 3). Among those without COPD, 117 were in group 1, 280 were in group 2, 64 were in group 3, and 201 were in group 4. ST differed significantly across groups, with group 4 (4.0 (1.0–20.0)) showing higher values, *p* = 0.003, adjusted for multiple comparisons (Table 4). In a multivariable linear regression model including COPD, OSA, PLMS, age, and sex, both COPD and OSA were independently associated with longer ST ⩽ 88%. COPD (Figure 2), increased ST by +46.4 min (95% CI 23.6–69.2, *p* < 0.001) and OSA by +10.5 min (95% CI 2.6–18.5, *p* = 0.009). PLMS alone was not significant. Interaction analyses revealed that COPD × OSA had a significant negative effect (β = −35.9, *p* = 0.021), suggesting less-than-additive combined effects. By contrast, a three-way interaction of COPD × OSA × PLMS was positive (β = +49.1, *p* = 0.036), indicating that participants with all three conditions had the greatest burden of hypoxemia.

**Table 2.** Comparison of sleep architecture and respiratory indices (mean ± SD for normally distributed, median (IQR) for skewed variables).

Parameter	COPD 0 Mean ± SD	COPD 1 Mean ± SD	*p*-Value (adjusted for age)
Total sleep time	300.249 ± 91.359	277.729 ± 94.902	0.841
Sleep latency	30.105 ± 36.063	29.445 ± 29.814	0.651
REM latency	168.335 ± 96.359	182.432 ± 90.483	0.499
Wake after sleep onset	88.522 ± 85.147	96.855 ± 70.745	0.7
Sleep efficiency	71.312 ± 20.050	67.665 ± 21.550	0.808
Number of awakenings	20.558 ± 12.003	19.458 ± 9.020	0.361
REM arousal index	10.640 ± 16.859	11.523 ± 22.048	0.94
Minimum saturation	82.415 ± 7.014	80.312 ± 6.264	0.102
Mean saturation	93.264 ± 2.215	91.104 ± 2.800	0.001*
Percent of N1	5.893 ± 4.299	5.383 ± 3.461	0.65
Percent of N2	44.825 ± 15.707	45.508 ± 16.680	0.534
Percent of N3	9.484 ± 7.530	7.419 ± 6.257	0.928
Percent of REM	11.111 ± 7.689	9.346 ± 8.365	0.617
Central apnea index	1.056 ± 3.466	2.012 ± 5.709	0.167
AHI, median (IQR)	10.2 (3.8–27.6)	12.1 (4.5–33.3)	0.590
OSA frequency (AHI ⩾5)	472 (71.2%)	34 (70.8%)	0.97
OSA severity: mild/mod/severe	205/152/115	14/11/9	–
PLMI, median (IQR)	9.0 (2.5–26.0)	13.5 (4.0–38.0)	0.319
ST < 88% (min), median (IQR)	2.0 (0.0–8.0)	27.0 (7.5–68.0)	0.001*

AHI, apnea–hypopnea index; OSA, obstructive sleep apnea; PLMI, periodic limb movement index; ST, saturation time.

* statistically significant.

**Table 3.** Saturation time ⩽88% in patients with COPD (COPD 1) ⩽88% stratified by OSA and PLMS status (median (IQR)).

Subgroup	*N*	ST ⩽88% (min), median (IQR)	*p*-Value vs Group 1
Group 1 (No OSA/PLMS)	12	10.5 (2.0–45.0)	Ref
Group 2 (OSA only)	11	20.0 (8.0–40.0)	0.18
Group 3 (PLMS only)	8	15.0 (4.0–62.0)	0.22
Group 4 (OSA + PLMS)	17	38.0 (18.0–79.0)	<0.01*

ANCOVA adjusted for age, Tukey correction.

OSA, obstructive sleep apnea; PLMI, periodic limb movement index; ST, saturation time.

* statistically significant.

**Table 4.** Saturation time ⩽88% in patients without COPD (COPD 0) stratified by OSA and PLMS status (median (IQR)).

Subgroup	*N*	ST ⩽88% (min), median (IQR)	*p*-Value vs Group 1
Group 1 (No OSA/PLMS)	117	0.0 (0.0–2.0)	Ref
Group 2 (OSA only)	280	3.0 (0.5–12.0)	**<0.05**
Group 3 (PLMS only)	64	1.5 (0.0–6.0)	0.09
Group 4 (OSA + PLMS)	201	4.0 (1.0–20.0)	<0.01*

ANCOVA adjusted for age, Tukey correction.

OSA, obstructive sleep apnea; PLMS, periodic limb movements of sleep; ST, saturation time.

* statistically significant.

In Supplemental analyses (Supplemental Table S1), two-way ANOVA stratified by COPD status showed that OSA was significantly associated with longer ST ⩽88% in non-COPD participants (*p* = 0.0006). By contrast, PLMS and the OSA × PLMS interaction were not significant. In COPD participants, neither OSA, PLMS, nor their interaction reached significance. By contrast, multivariable regression across the entire cohort (Table 5) demonstrated that both COPD and OSA independently increased ST, with a significant negative COPD × OSA interaction (p = 0.021) indicating a less-than-additive combined effect. In addition, a significant three-way COPD × OSA × PLMS interaction (*p* = 0.036) was observed, showing that participants with all three conditions had the greatest desaturation burden.

**Table 5.** Multivariable linear regression of ST ⩽88% dependent variable: ST (minutes).

Predictor	β (Coef, min)	95% CI	*p*-Value
COPD	**+46.4**	23.6–69.2	<0.001*
OSA	**+10.5**	2.6–18.5	0.009*
PLMS	+5.6	−5.9–17.0	0.341
COPD × OSA	−**35.9**	−66.5–-5.4	0.021*
COPD × PLMS	−8.3	−44.9–28.3	0.655
OSA × PLMS	−1.9	−15.0–11.2	0.773
**COPD × OSA × PLMS**	**+49.1**	3.2–95.0	0.036*
Age (years)	−0.04	−0.20–0.11	0.583
Sex (male)	−0.48	−6.0–5.1	0.866

Adjusted for age and sex.

COPD, chronic obstructive pulmonary disease; OSA, obstructive sleep apnea; PLMS, periodic limb movements of sleep.

* statistically significant.

**Figure 3.**
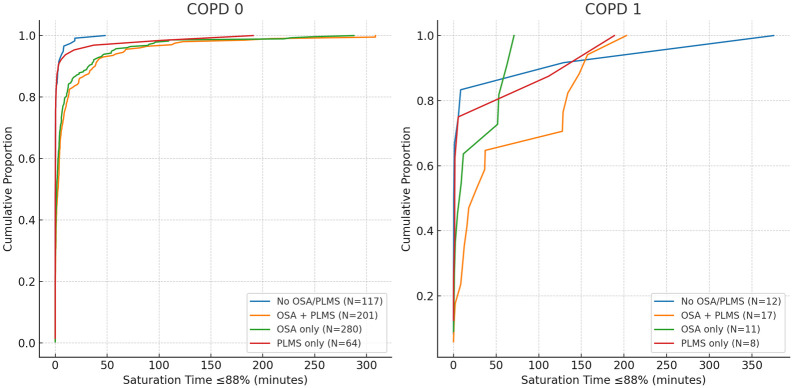
Cumulative distribution of saturation time spent at or below 88% (ST). This figure illustrates the cumulative distribution of saturation time (ST) below 88% among OSA/PLMS groups, stratified by COPD status. The X-axis represents saturation time in minutes, and the *Y*-axis shows the cumulative proportion of patients. Each line represents one of the four OSA/PLMS groups: Group 1 (no OSA or PLMS), Group 2 (OSA only), Group 3 (PLMS only), and Group 4 (both OSA and PLMS). The left panel corresponds to patients without COPD (COPD 0), while the right panel corresponds to patients with COPD (COPD 1). This plot highlights the variability in oxygen desaturation time distributions across the groups and the pronounced effect in COPD 1 patients. COPD, chronic obstructive pulmonary disease; OSA, obstructive sleep apnea; PLMS, periodic limb movements of sleep.

Cumulative distribution functions (CDFs) were plotted for ST (Figure 3), stratified by OSA/PLMS groups within COPD 0 and COPD 1.

## Discussion

In this study, we compared sleep architecture, respiratory indices, and oxygen saturation metrics between COPD and non-COPD patients and evaluated subgroups of patients without OSA/PLMS, with OSA alone, with PLMS alone, and with both OSA and PLMS. Our findings provide insights into the interplay between COPD and sleep disorders, highlighting distinct patterns of oxygen desaturation, particularly in the context of comorbid elevated index of PLMS and OSA. Particularly, the findings highlight complementary aspects of how COPD, OSA, and PLMS contribute to nocturnal hypoxemia. The two-way ANOVA indicated that in participants without COPD, OSA was a clear driver of desaturation, whereas PLMS had no independent effect. In those with COPD, however, neither OSA nor PLMS significantly influenced hypoxemia, suggesting that the underlying pulmonary impairment is the dominant determinant of oxygen desaturation in this group. The regression analysis provided additional nuance, showing that while COPD and OSA each independently prolonged desaturation time, their combined effect was less than additive, consistent with overlapping mechanisms of hypoxemia. Importantly, the significant three-way interaction demonstrated that the coexistence of COPD, OSA, and PLMS confers the highest risk for nocturnal hypoxemia, suggesting a synergistic effect when all three disorders are present. Together, these analyses indicate that the contribution of OSA is most evident in non-COPD patients, is diminished in COPD alone, but may re-emerge in a clinically meaningful way when PLMS co-occurs with both conditions.

In general, it is expected that patients with COPD spend more time with saturation below 88% compared to those without COPD.^12^ Sleep architecture was largely similar between those with COPD and those without. This is consistent with studies suggesting that while COPD affects respiratory indices, its direct impact on sleep structure may be more subtle or mediated through comorbid conditions like OSA.

When stratified by subgroups according to the presence of OSA or an elevated index of PLMS, differences in desaturation time were noted. Among COPD patients, those in Group 4 (with both OSA and PLMS) had the highest ST below 88%, suggesting that the combined burden of these disorders may exacerbate nocturnal hypoxemia. Similarly, in non-COPD patients, those with both OSA and PLMS showed more oxygen desaturation than those with either condition alone, though to a lesser extent than in COPD patients. These findings suggest the effect of OSA and PLMS on oxygen desaturation, which is amplified in the presence of COPD. This is evident in the CDF plots in Figure 3.

OSA contributes to intermittent hypoxemia through repeated upper airway obstructions, leading to ventilation-perfusion mismatch and increased intrathoracic pressure, both of which are exacerbated by the reduced pulmonary reserve in COPD.^13^ PLMS are characterized by repetitive leg movements associated with transient cortical arousals, each accompanied by surges in sympathetic nervous system activity.^14^ These surges lead to acute elevations in heart rate, blood pressure, and peripheral vasoconstriction,^15^ increasing cardiovascular oxygen demand, which may worsen ventilation–perfusion mismatch in patients with COPD, whose gas exchange is already compromised. Over time, this mechanism can exacerbate nocturnal hypoxemia. Furthermore, chronic intermittent arousals from PLMS promote sustained sympathetic activation, which can compound the hypoxemic stress by causing pulmonary vasoconstriction and inflammatory responses. Moreover, heightened sympathetic tone has been linked to systemic inflammation and endothelial dysfunction.^16^ both of which could exacerbate nocturnal hypoxemia in COPD. A previous study on patients with decreased pulmonary reserve has shown that PLMS were correlated with hypoxemia.^17–19^

Combined, these conditions are likely to act synergistically to amplify nocturnal hypoxemia in overlap syndrome patients. Furthermore, chronic inflammation^20^ and heightened sympathetic activity, common in both OSA and PLMS,^21,22^ can be compounded in COPD, contributing to worsening oxygenation.

While OSA-related airway collapse causes direct oxygen desaturation, PLMS-related arousals cause surges in sympathetic output and sleep fragmentation that hinder recovery and stabilize breathing, together worsening overall hypoxemia. However, we acknowledge that the association could partly reflect reverse causation: chronic hypoxemia in COPD might itself trigger more frequent PLMS episodes (e.g., via chemoreceptor stimulation or neural mechanisms). Our cross-sectional study cannot definitively establish causality, and both explanations (PLMS exacerbating hypoxemia and hypoxemia precipitating PLMS) are plausible.

The findings have important clinical implications. First, the significantly higher saturation time below 88% in patients with COPD with comorbid OSA and abnormal index of PLMS may need special attention to saturation when on continuous positive airway pressure (CPAP), and supplemental oxygen therapy may be needed.

## Strengths and limitations

A major strength of this study is the detailed stratification of patients by COPD status and OSA/PLMS subgroups, as well as the use of CDFs providing a robust visualization of saturation time differences across groups.

However, this work has some limitations, including the following. First, as a retrospective observational study, it cannot establish causal relationships. Second, we did not adjust for certain potential confounders or comorbid conditions. Other medical factors, such as coexisting cardiovascular disease, heart failure, or metabolic disorders, as well as medications and treatments (e.g., the use of nocturnal supplemental oxygen or CPAP adherence), were not accounted for and could influence both PLMS and oxygenation.

In addition, the relatively small sample size in some subgroups (particularly the COPD with PLMS-only subgroup) may have limited our power to detect subtle differences in certain sleep parameters. Future studies with larger, prospective cohorts are warranted to validate these findings and further elucidate the underlying mechanisms by which PLMS and OSA interact in COPD. Such studies should also incorporate a more comprehensive assessment of confounding variables (comorbidities and treatments) and could explore whether interventions targeting PLMS reduce hypoxemic burden in COPD overlap syndrome patients.

## Conclusion

This study demonstrates that COPD is associated with prolonged nocturnal hypoxemia, particularly when combined with OSA. Multivariable analysis confirmed that both COPD and OSA significantly contribute to longer saturation times ⩽88%, while PLMS alone was not a major predictor. However, interaction effects revealed that patients with coexisting COPD, OSA, and PLMS experienced the greatest burden of nocturnal hypoxemia. These findings emphasize the impact of COPD and sleep disorders on oxygen desaturation, highlighting the need for comprehensive evaluation and management of OSA and PLMS in patients with COPD. Future research should assess targeted interventions to mitigate the combined effects of these conditions on nocturnal hypoxemia and related outcomes.

## Supplemental Material

sj-doc-1-tar-10.1177_17534666251380431 – Supplemental material for Nocturnal hypoxemia in COPD: the amplifying effect of comorbid OSA and PLMS on oxygen desaturation Supplemental material, sj-doc-1-tar-10.1177_17534666251380431 for Nocturnal hypoxemia in COPD: the amplifying effect of comorbid OSA and PLMS on oxygen desaturation by Viraj Jain, Harshill Modi, Moon Park, Anil Ghimire and Lourdes M. DelRosso in Therapeutic Advances in Respiratory Disease

sj-docx-1-tar-10.1177_17534666251380431 – Supplemental material for Nocturnal hypoxemia in COPD: the amplifying effect of comorbid OSA and PLMS on oxygen desaturation Supplemental material, sj-docx-1-tar-10.1177_17534666251380431 for Nocturnal hypoxemia in COPD: the amplifying effect of comorbid OSA and PLMS on oxygen desaturation by Viraj Jain, Harshill Modi, Moon Park, Anil Ghimire and Lourdes M. DelRosso in Therapeutic Advances in Respiratory Disease

## Declarations

**Ethics approval and consent to participate:** This study was approved by the Community Health System Institutional Review Board (IRB 2023052). Because the study involved a retrospective review of polysomnography data, the IRB waived the requirement for additional informed consent. All patients provided consent to undergo polysomnography as part of their clinical care. The requirement for research-specific informed consent was waived by the IRB due to the retrospective nature of the study.

**Consent for publication:** Not applicable. The manuscript does not contain any individual person’s identifiable data (including images, videos, or personal details).

**Availability of data and materials:** The data that support the findings of this study are not publicly available due to Community Health System organizational policies, but are available from the corresponding author on reasonable request, subject to institutional approval.
