# Supplementary material for: Nocturnal hypoxemia in COPD: the amplifying effect of comorbid OSA and PLMS on oxygen desaturation
Source: Ther Adv Respir Dis. 2025 Sep 17;19:17534666251380431. doi: 10.1177/17534666251380431 (PMC12444068; doi:10.1177/17534666251380431)
Supplement: sj-docx-1-tar-10.1177_17534666251380431 – Supplemental material for Nocturnal hypoxemia in COPD: the amplifying effect of comorbid OSA and PLMS on oxygen desaturation [file sj-docx-1-tar-10.1177_17534666251380431.docx]

Supplementary Table S1. Two-way ANOVA: Effects of OSA and PLMS on ST ≤88%

| **Factor** | **COPD 0 F(df)** | **p-value** | **COPD 1 F(df)** | **p-value** |
| --- | --- | --- | --- | --- |
| OSA | F(1,659) = 11.8 | **0.0006** | F(1,43) = 0.04 | 0.852 |
| PLMS | F(1,659) = 2.1 | 0.151 | F(1,43) = 1.39 | 0.246 |
| OSA × PLMS | F(1,659) = 0.09 | 0.771 | F(1,43) = 1.04 | 0.314 |
